# The impact of close surveillance on pregnancy outcome among women with a prior history of antepartum complications attributed to thrombosis: a cohort study

**DOI:** 10.1186/1477-7827-6-55

**Published:** 2008-11-21

**Authors:** Raed Salim, Tali Czarnowicki, Zohar Nachum, Eliezer Shalev

**Affiliations:** 1 Department of Obstetrics and Gynecology, HaEmek Medical Center, Afula, Israel; 2 Rappaport Faculty of Medicine, Technion, Haifa, Israel

## Abstract

**Background:**

There is limited evidence, so far, as to the optimal management of women with a prior obstetric history of antepartum complications attributed to thrombosis. We aimed to investigate the contribution of close antepartum surveillance on pregnancy outcome among women with prior antepartum complications attributed to thrombosis.

**Methods:**

The study was conducted on all women who were delivered, conceived and delivered again between January 2000 and January 2006 at a university teaching hospital. Women included were managed in previous pregnancy at a low risk setting and had unpredicted antepartum complications occurring at a gestational age of 23 weeks or more. Antepartum complications considered were intrauterine fetal death, neonates who were small for gestational age, severe pre-eclampsia and placental abruption. All women were tested for the presence of thrombophilia after delivery. In the following pregnancy, only women found to have any thrombophilia (thrombophilic group) were treated with enoxaparin. Both the thrombophilic group and the non-thrombophilic group (tested negatively for thrombophilia) were managed and observed closely at our high-risk pregnancy clinic.

**Results:**

Ninety-seven women, who conceived at least once after the diagnosis of the relevant antepartum complications, were included in this study. Forty-nine had any thrombophilia and 48 tested negatively. Composite antepartum complications (all antepartum complications considered) were reduced significantly after close antepartum surveillance in both groups. Mean birth weight and mean gestational age improved significantly and were comparable between the groups.

**Conclusion:**

Close antepartum surveillance may contribute to improvement in the perinatal outcomes of women with prior antepartum complications attributed to thrombosis.

## Background

Severe pre-eclampsia, intrauterine growth restriction, intrauterine fetal death, and placental abruption affect 8% of pregnant women and collectively contribute to the largest proportion of maternal and fetal mortality and morbidity [1]. Evidence has accumulated to suggest that these antepartum complications could have a common thrombogenic basis that manifested in the placenta as placental infarction, perivillous fibrin deposition, intervillous thrombosis and placental floor infarction [2,3]. Although acquired and inherited thrombophilia had been reported to increase significantly the incidence of antepartum complications attributed to thrombosis [4-8], Mousa et al reported that no different or specific histological pattern could be identified among women with antepartum complications when thrombophilia positive and thrombophilia negative groups were compared [3].

There is limited evidence, so far, as to the optimal management of women with a prior obstetric history of antepartum complications attributed to thrombosis. Since these women are considered at a high risk of an adverse maternal or fetal outcome in subsequent pregnancies [1], we aim in this study to investigate the possible beneficial effect of close antepartum surveillance among these women who conceived and delivered again at our institution.

## Methods

Women who had a previous pregnancy with antepartum complications occurring at a gestational age of 23 weeks or more were followed and tested for thrombophilia. Antepartum complications considered were unexplained intrauterine fetal death, neonates who were small for gestational age (SGA), severe pre-eclampsia and placental abruption. SGA was defined as birth weight below the 10^th ^percentile. Severe pre-eclampsia was defined as blood pressure measuring above 140/90 combined with proteinuria (300 mg/24 h) and accompanied with one or more of the following complications: worsening blood pressure to a level above 160/110, HELLP syndrome (hemolysis, low platelet count and elevated liver enzymes), persistent severe headache or visual disturbances, persistent severe epigastric pain, nausea and vomiting, eclampsia, pulmonary edema, oligouria (urine output less than 500 cc/24 h), proteinuria of 5 g or more per 24 h and fetal growth restriction (below the 5^th ^percentile). The study was conducted at a university teaching hospital on all women who were delivered, tested for thrombophilia after delivery, conceived and delivered again between January 2000 and January 2006. Women were tested for the mutation of guanine to adenine at nucleotide 1691 in the factor V gene (factor V Leiden), the mutation of cytosine to thymine at nucleotide 677 in the gene encoding methylenetetrahydrofolate reductase, and the mutation of guanine to adenine at nucleotide 20210 in the prothrombin gene. Women were also tested for protein C, protein S and antithrombin III deficiency; and the presence of antiphospholipid antibodies (lupus anticoagulant or anticardiolipin antibodies) and the results were confirmed in a second assay. Blood tests were done six weeks or more after the previous delivery. Women who had a previous pregnancy with antepartum complications that could be attributed to multiple gestations, having fetuses with major congenital anomalies or chromosomal abnormalities, fetal infection, chorioamnionitis, hydrops fetalis and diabetes mellitus were excluded. Only women found to have any thrombophilia (thrombophilic group) were treated for the first time, in the following pregnancy, with the low molecular weight heparin (LMWH), enoxaparin (Lovenox, Aventis, France). Enoxaparin was started after detection of fetal heart activity in the 1^st ^trimester throughout gestation, and six weeks into the post-partum period. The enoxaparin dose was 40 mg once daily in women with a solitary thrombophilia and 80 mg daily (40 mg every 12 h) in women with combined thrombophilia. This higher dose was used based upon the significantly higher risk for pregnancy loss and stillbirth in women with combined thrombophilia [9]. Aspirin at a dose of 100 mg (Aspe'gic nourrissons, Sanofi-Synthe'labo, France) daily was given in addition to enoxaparin to women with antiphospholipid antibodies. Additionally, all women received 5 mg folic acid daily. Both the thrombophilic group and the non-thrombophilic group were managed and observed closely at our high-risk pregnancy clinic during the study period. Surveillance of both groups included weekly blood pressure measurements and sonographic estimates of fetal weight at 4 weeks intervals. When fetal growth restriction (below the 10^th ^percentile) was detected, sonographic fetal weight estimates were done every two weeks. In addition, non-stress testing (NST) twice weekly, umbilical artery Doppler and biophysical profile once weekly were also performed. Women detected to have fetal growth restriction with a reassuring fetal status were allowed to continue surveillance until term (37 weeks). At 37 – 38 weeks, induction of labor was considered. Women with a previous intrauterine fetal death had a weekly NST starting a week before the gestational age when fetal death was detected in the prior pregnancy. These women were induced between 38 – 39 weeks. Women who developed pre-eclampsia or placental abruption were managed according to the severity of the disease, fetal status and gestational age. Women were asked about bleeding episodes, signs of thrombosis, and symptoms of bone pain. Levels of platelets were followed monthly.

We compared perinatal outcomes within and between the groups before and after close surveillance. Perinatal outcomes included the reappearance of one or more of the thrombophilic complications considered (fetal death, SGA, severe pre-eclampsia and placental abruption), neonatal birth weight and Apgar score. Other parameters collected were maternal age and ethnicity. The local Institutional Review Board approved the study.

### Statistical analysis

Data analysis was performed using the SPSS 14 statistical package (SPSS, Chicago, IL). Comparison of binary variables between thrombophilic and non-thrombophilic groups, were analyzed by Chi-square test or Fisher's exact test as appropriate. T test or Mann Withney test were used to compare continuous variables between the two independent groups. To assess the influence of treatment on the variables in each group, McNemar, Paired T test or Wilcoxon signed ranks test were applied as appropriate. Proportion of live born neonates per woman was calculated as number of live born neonates divided by number of births per woman. The same calculation was made regarding the proportion of preterm deliveries and complicated pregnancies per woman. A p value < 0.05 was considered significant.

## Results

Ninety-seven women, who conceived at least once after the diagnosis of the relevant antepartum complications, were included in this study. Of all the 97 women, 49 had any thrombophilia and 48 tested negatively for thrombophilia. Of all the 97 women, 54 were Arabs and 43 were Jews. Of the 54 Arab women, 34 (63%) had thrombophilia compared to 15 out of 43 (35%) Jews (p = 0.008, OR 3.2, 95% CI 1.4 to 7.3). The thrombophilic profile of the thrombophilic group is presented in table 1. Thirty-nine women had a solitary thrombophilic defect, while 10 women had a combined thrombophia. Factor V Leiden (FVL) was the most common thrombophilic defect, presented in 16 out of 39 women (41%) as a solitary finding and in 8 of 10 women (80%) as part of a combined defect.

**Table 1:** The thrombophilic profile of the 49 women who belonged to the thrombophilic group.

Single thrombophilia	No.	Combined thrombophilia	No.
Factor V Homozygote	1	Factor V Heterozygote + MTHFR homozygote	4
Factor V Heterozygote	15	Factor V Heterozygote + Protein S deficiency	3
Factor II Homozygote	0	Factor V Homozygote + Protein S deficiency	1
Factor II Heterozygote	5	ANT III deficiency + MTHFR homozygote	1
MTHFR homozygote	7	Protein S deficiency + Protein C deficiency	1
Protein C deficiency	0		
Protein S deficiency	3		
ANT III deficiency	0		
Antiphospholepid antibodies	8		

MTHFR = methylenetetrahydrofolate reductase.

ANT = antithrombin.

Among the thrombophilic group, the number of women with at least one live born neonate was significantly increased, and the composite antepartum complications (all antepartum complications considered) were reduced significantly after close surveillance (table 2). In addition, the incidence of antepartum complications per woman, before and after close surveillance combined with LMWH treatment, was also significantly reduced (table 3). Gestational age at delivery was 33 weeks (+/- 5.9) compared with 37.3 weeks (+/- 3.4), (p < 0.01), and the mean birth weight among women who had live born neonates before and after close surveillance was 2353 g (+/- 756) and 2869 g (+/- 641) respectively (p < 0.01).

**Table 2:** Comparison of perinatal outcomes within the thrombophilic and non-thrombophilic groups before and after close surveillance.

	Thrombophilic group (N = 49)			Non-thrombophilic group (N = 48)		
Number of women with at least one	Before close surveillance N (%)	After close surveillance N (%)	P*	Before close surveillance N (%)	After close surveillance N (%)	P*
Live born neonate	35 (71)	46 (94)	0.004	40 (83)	44(92)	0.1
Preterm delivery	20 (41)	5 (10)	0.001	27 (56)	16 (33)	0.052
Complicated pregnancies	49 (100)	6 (12)	<0.001	48 (100)	19 (40)	<0.001
SGA	21 (43)	5 (10)	<0.001	21 (44)	12 (25)	0.06
IUFD	25 (51)	2 (4)	<0.001	20 (42)	3 (6)	<0.001
Severe pre-eclampsia	9 (18)	1 (2)	0.02	10 (21)	4 (8)	0.1
Placental abruption	18 (37)	1 (2)	<0.001	18 (38)	5 (10)	0.004

* McNemar test

Complicated pregnancies = the reappearance of one or more of the thrombophilic complications considered (fetal death, SGA, severe pre-eclampsia and placental abruption)

SGA = neonate small for gestational age

IUFD = intrauterine fetal death

**Table 3:** Comparison of the proportion of complications per woman within the thrombophilic and non-thrombophilic groups before and after close surveillance.

	Thrombophilic group (N = 49)			Non-thrombophilic group (N = 48)		
Proportion, per woman, of	Before close surveillance	After close surveillance	P*	Before close surveillance	After close surveillance	P*
Live born neonate	63 (43) [80]	96 (20) [100]	<0.001	74 (38) [100]	96 (21) [100]	0.002
Preterm deliveries	30 (42) [0]	9 (29) [0]	0.01	42 (44) [33]	32 (46) [0]	0.4
Complicated pregnancies	70 (32) [67]	11 (31) [0]	<0.001	63 (35) [50]	35 (46) [0]	0.004

The data are presented as mean proportion, (standard deviation) and [median]

* Wilcoxon Signed Rank Test

Among the non-thrombophilic group, the number of women with at least one live neonate was not increased significantly following close surveillance; however, the incidence of live born neonates per woman, before and after close surveillance, was significantly increased (table 2 and 3). Composite antepartum complications were reduced significantly after close surveillance. The number of women who had severe pre-eclampsia or delivered small for age neonates did not differ significantly (table 2 and 3). Gestational age at delivery was 32.4 weeks (+/- 4.8) compared with 36.4 weeks (+/- 3.9) after close surveillance (p < 0.01). The mean birth weight among women who had live born neonates before and after close surveillance was 2213 g (+/- 918) and 2592 g (+/- 820) respectively (p = 0.03). Before close surveillance, all the parameters studied were comparable between the thrombophilic group and the non-thrombophilic group (table 4). After close surveillance, beside the incidence of preterm deliveries, each individual antepartum complications studied was comparable between the groups (table 4). Gestational age and birth weight did not differ between the groups before and after close surveillance. Among the thrombophilic group, 19.4% of women delivered by cesarean section compared to 24.5% among the non-thrombophilic group after close surveillance (p = 0.5). Of all the vaginal deliveries, 28% and 40% were induced in the thrombophilic group and the non-thrombophilic group respectively (p = 0.3). Three cases of postpartum bleedings were noticed only within the thrombophilic group (p = 0.3). Among all live neonates, none had an Apgar score of less than 7 at 5 minutes in either group.

**Table 4:** Comparison between perinatal outcomes of thrombophilic and non-thrombophilic groups before and after close surveillance

	Before close surveillance			After close surveillance		
	Thrombophilic group (N = 49)	Non-thrombophilic group (N = 48)	*P*	Thrombophilic group (N = 49)	Non-thrombophilic group (N = 48)	*P*
Proportion, per woman, of						
Live born neonate*	63 (43) [80]	74 (38) [100]	0.2†	96 (20) [100]	96 (21) [100]	0.9†
Preterm deliveries*	30 (42) [0]	42 (44) [33]	0.1†	9 (29) [0]	32 (46) [0]	0.005†
Number of women who delivered at least one						
SGA (%)	21 (43)	21 (44)	0.9‡	5 (10)	12 (25)	0.06‡
IUFD (%)	25 (51)	20 (42)	0.4‡	2 (4)	3 (6)	0.7§
Severe pre-eclampsia (%)	9 (18)	10 (21)	0.8‡	1 (2)	4 (8)	0.2§
Placental abruption (%)	18 (37)	18 (38)	0.9‡	1 (2)	5 (10)	0.1§

* The data are presented as mean proportion, (standard deviation) and [median]

† Mann Withney test

‡ Chi-square test

§Fisher's exact test

SGA = neonate small for gestational age

IUFD = intrauterine fetal death.

We did not observe any case of heparin-induced thrombocytopenia among the 49 women treated with LMWH. Likewise, there were no clinical manifestations of bone pain or bleeding complications related to LMWH treatment among the women.

## Discussion

The present report demonstrates that close antepartum surveillance at specialized clinics may contribute to improvement in the perinatal outcomes of women with prior antepartum complications attributed to thrombosis regardless of the presence of thrombophilia detected by our testing methods. Among these women, the incidence of any thrombophilic defect was 51%, which is, similar to that reported in the literature [10]. The FVL mutation was the most common type of thrombophilia, accounting for 49% of all the thrombophilic defects. The incidence of thrombophilia was significantly higher among Arabs compared to Jews. The high incidence of thrombophilia among Israel's Arabs compared to Jews maybe explained by the high rate of consanguinity reported in that population [11,12].

The present report demonstrates that among women with antepartum complications who were found to have inherited or acquired thrombophilia, close surveillance combined with antithrombotic therapy in subsequent gestations, resulted in a significant improvement in perinatal outcome, compared to the previous gestations. Contradictory reports are present in the literature regarding the effect and role of LMWH treatment on thrombophilic women with a history of pre-eclampsia, intrauterine growth restriction, placental abruption and fetal loss [13-19]. However, in view of the potential severe consequences of thrombophilia in pregnancy, several reports have recommended the use of anticoagulant therapy in the following pregnancies [20,21]. A main issue which raised in this report is that these pregnancies, once diagnosed, are usually managed under close surveillance and, still, none of these studies had isolated the role of close antepartum surveillance on pregnancy outcome among these women. Thus it is still to be determined whether LMWH alone, close surveillance or both is responsible for the better outcome.

In this study, 49% of the women who had antepartum complications were tested negatively for thrombophilia and therefore were not treated with LMWH. After close surveillance alone in subsequent gestations, a significant improvement was observed in composite fetal outcome compared to previous gestations. Nevertheless, and probably expected, the number of women who had severe pre-eclampsia or delivered SGA neonates did not differ significantly with close surveillance alone. Close surveillance alone, decreased significantly the incidence of IUFDs per woman. Moreover, gestational age and birth weight were both improved, probably due to the opportunity of prolonging the gestational age under close surveillance. When comparing perinatal outcome between the groups, antepartum complications were reduced significantly in both groups after close surveillance. Moreover, gestational age at delivery, birth weight and the incidence of live born neonate per woman were comparable between the groups.

Comparing the thrombophilic and the non-thrombophilic group, who tested negatively for the thrombophilic defects studied, might seem ill advised. Nevertheless, both groups share probably the same uteroplacental histopathology. Mousa et al reported that no specific histological pattern could be identified among women with antepartum complications when thrombophilia positive and thrombophilia negative groups were compared and concluded that a poor correlation exists between thrombophilia status and pathological changes of the placenta in women with antepartum complications [3]. Moreover, it cannot be ruled out that the non-thrombophilic group possesses thrombophilic defects that are unknown or were not studied in this report [22]. We did not stratify the obtained results with the level of fasting total homocysteine, because all women were taking folic acid from at least 1 month before conception [23].

Moreover, we did not examine the maternal plasma levels of coagulation factor VIII, which had been reported to be associated with an increased risk for recurrent early pregnancy loss [24], or for protein Z deficiency and positive antiprotein Z antibodies, which had been described to increase the severity of the prothrombotic phenotype of FVL [25]. Whether the non-thrombophilic group has an unrecognized thrombophilia or not, close surveillance without any other treatment improved their perinatal outcomes.

## Conclusion

Although, and due to ethical issues, the women in both groups were not randomized and all got the same close antepartum surveillance, this study reveals that close surveillance at a specialized clinic may improve the perinatal outcomes of both thrombophilic and non-thrombophilic women with prior antepartum complications attributed to thrombosis. However, this study dose not separate the effect of LMWH and close antepartum surveillance on pregnancy outcome among the thrombophilic group and it still needs to be determined whether the additional treatment of LMWH is better than close surveillance alone. While this study cannot answer the latter issue, its results may be considered an intermediate step in allowing placebo to be compared to LMWH during pregnancy in thrombophilic women with previous placental dysfunction.

## Competing interests

The authors declare that they have no competing interests.

## Authors' contributions

RS and ES designed the research, supervised the data collection, assisted in the statistical analysis, and wrote the manuscript. TC and ZN collected and maintained the data and assisted in writing the manuscript. All authors read and approved the final manuscript.
